# Achieving PSA < 0.2 ng/ml before Radiation Therapy Is a Strong Predictor of Treatment Success in Patients with High-Risk Locally Advanced Prostate Cancer

**DOI:** 10.1155/2019/4050352

**Published:** 2019-10-17

**Authors:** Akira Kazama, Toshihiro Saito, Keisuke Takeda, Kazuhiro Kobayashi, Toshiki Tanikawa, Ayae Kanemoto, Fumio Ayukawa, Yasuo Matsumoto, Tadashi Sugita, Noboru Hara, Yoshihiko Tomita

**Affiliations:** ^1^Department of Urology, Niigata Cancer Center Hospital, 2-15-3, Kawagishicho, Chuo-ku, Niigata 951-8133, Japan; ^2^Division of Urology, Department of Molecular Oncology, Niigata University Graduate School of Medical and Dental Sciences, 1-757, Asahimachi-dori, Chuo-ku, Niigata 951-8510, Japan; ^3^Department of Radiation Therapy, Niigata Cancer Center Hospital, 2-15-3, Kawagishicho, Chuo-ku, Niigata 951-8133, Japan

## Abstract

**Background:**

To predict long-term treatment outcome of radiation therapy (RT) plus androgen deprivation therapy (ADT) for high-risk locally advanced prostate cancer.

**Methods:**

In total, 204 patients with the National Comprehensive Cancer Network (NCCN) high risk locally advanced prostate cancer (PSA > 20 ng/ml, Gleason score ≧ 8, clinical T stage ≧ 3a) were treated with definitive RT with ADT. Median follow up period was 113 months (IQR: 95–128). Median neoadjuvant ADT and total ADT duration were 7 months (IQR: 6–10) and 27 months (IQR: 14–38), respectively.

**Results:**

PSA recurrence-free survival (PSA-RFS), cancer specific survival (CSS), and overall survival (OS) rates at 5 years were 84.1%, 98.5%, and 93.6%, respectively, and 67.9%, 91.2%, and 78.1%, respectively, at 10 years. Pre-RT PSA less than 0.2 ng/ml was associated with superior outcomes of PSA-RFS (HR = 0.42, 95% CI: 0.25–0.70, *p* = 0.001), CSS (HR = 0.27, 95% CI: 0.09–0.82, *p* = 0.013), and OS (HR = 0.48, 95% CI: 0.26–0.91, *p* = 0.021). On multivariate analysis, age (≥70 y.o.) and pre-RT PSA (≥0.2 ng/ml) were factors predictive of poorer OS (*p* = 0.032) , but iPSA, T stage, Gleason score, number of NCCN high-risk criteria, a combination with anti-androgen therapy and neoadjuvant ADT duration were not predictive of treatment outcome.

**Conclusion:**

In patient with high-risk prostate cancer, RT plus ADT achieved good oncologic outcomes. PSA < 0.2 ng/ml before radiation therapy is a strong independent predictor for long overall survival.

## 1. Introduction

In recent years, the National Comprehensive Cancer Network (NCCN) guidelines subdivided the risk classification of prostate cancer (PCa). According to the guidelines, radical prostatectomy or radiation therapy (RT) with androgen deprivation therapy is recommended for high-risk prostate cancer, initial PSA (iPSA) > 20 ng/ml, Gleason score ≥ 8 or locally advanced (≥T3a). Additionally, RT should be performed in conjunction with 4–6 months of neoadjuvant ADT (NADT) and 2–3 years adjuvant ADT (AADT) in patients with high-risk PCa. The efficacy of NADT was reported in several phase III clinical trials, RTOG86-10 [1]and TROG9601 [2]. In addition, the results of EORTC22863 [3],RTOG85-31 [4], and RTOG92-02 [5] indicated that patients with high-risk PCa who received AADT had longer overall survival.

In combination with definitive local therapy, the role played by ADT is considered as follows; in NADT, the irradiation field can be narrowed in accordance with reduced tumor and prostate volume, leading to higher treatment efficacy and less adverse events [6]. Moreover, concurrent extra-beam radiation therapy (EBRT) with ADT can enhance apoptosis, and synergistic effect can be expected [7]. AADT can bring about a suppressing efficacy against residual tumor and micro-metastasis. In addition, early responses to ADT are thought to be associated with better prognosis in men with prostate cancer.

Emerging reports indicated the association between oncologic outcomes and biochemical response after NADT. In recent meta-analysis showed prognostic value of low PSA level before EBRT (pre-RT PSA) for localized PCa [8]. The importance pre-RT PSA level after NADT in patients with high-risk PCa has been demonstrated in several studies [9–12].

In the present study, we reported the long-term treatment outcome of EBRT plus ADT in patients with high-risk prostate cancer. Furthermore, we investigated the relationship between PSA value before EBRT and patient outcomes.

## 2. Materials and Methods

Between January 2005 and December 2010, a total of 216 patients with NCCN high risk (PSA > 20 ng/ml, Gleason score ≥ 8, clinical T stage ≥ T3a) localized prostate cancer were treated with definitive EBRT (70 Gy/35 fr) and ADT combination. We investigated the clinicopathological features and oncologic outcome retrospectively. Of 216 patients, 12 were excluded due to early dropouts or insufficient data sets. The study protocol was reviewed and approved by the institutional review boards of the participating institutions.

The patients' medical records were accessed through the hospital charts and/or electronic medical records. Data collection included demographic data, PSA level at diagnosis (iPSA), clinical T stage, pathological findings, treatment, tumor response parameters, and data on patient outcomes. Clinical T stage was evaluated using magnetic resonance imaging in all patients.

All patients received NADT using luteinizing-hormone-releasing hormone (LHRH) agonist alone or in combination with the anti-androgens such as bicalutamide. Subsequently, they received definitive EBRT. Radiation therapy was delivered by 3-dimensional conformal radiation therapy (3DCRT) with 70 Gy. The clinical target volume included the prostate and seminal vesicles only and did not include regional pelvic nodes. Adjuvant ADT was continued for 24–36 months.

PSA levels after ADT and EBRT were principally obtained every 3 months the first 2 years and every 6 months thereafter. The lowest PSA before EBRT was defined as pre-RT PSA. Biochemical recurrence was defined as nadir PSA + 2.0 ng/ml based on the Phoenix definition [13]. Time to biochemical recurrence, time to cancer-specific death, and time to death were calculated from the start of ADT.

Table 1 shows patient and tumor characteristics. Median age was 71 y.o. (range: 57–83). The median initial PSA was 24.7 ng/ml (range: 1.9–282.5). The majority of the patients had clinical stage ≥ T3a (63.7%), and 64.2% of patients had Gleason score ≥ 8.119 patients (58.3%) had more than 2 factors of NCCN high-risk category. Median follow up period was 113 months (IQR: 95–128). Median NADT period and total ADT duration were 7 months (IQR: 6–10) and 27 months (IQR: 14–38), respectively. 142 patients (69.9%) had anti-androgen therapy. Median pre-RT PSA was 0.20 ng/ml (range: 0.04–1.2).

PSA recurrence-free survival (PAS-RFS), cancer-specific survival (CSS), and overall survival (OS) were estimated using the Kaplan–Meier method and tested using the log-rank test. Prognostic factors for overall survival were explored employing univariate and multivariate analyses with Cox-proportional hazard model. All statistical tests were two-sided, and *p*-value < 0.05 was considered significant. All statistical analysis was performed using the SPSS version 25.0.

## 3. Results

Of the entire cohort, 17 and 42 men died from prostate cancer and other causes, respectively. The overall survival rates at 5 and 10 years were 93.6% and 78.1%, respectively. Prostate cancer-specific survival at 5 and 10 years were 98.5% and 91.2%, respectively, and PSA recurrence-free survival rate at 5 and 10 years were 84.1%, and 67.9%, respectively.

Number of NCCN high risk criteria = 1 (HR = 0.42, 95% CI: 0.24–0.74, *p* = 0.003), and pre-RT PSA ≤ 0.2 ng/ml (HR = 0.42, 95% CI: 0.25–0.70, *p* = 0.021) were significantly associated with better PSA progression free-survival. Significant variables in the univariate analysis and possible prognostic factors such as iPSA, Gleason score, and anti-androgen use were additionally studied in a multivariate Cox regression analysis; however, none of them was risk factor for PSA recurrence (Table 2).

Age ≤ 70 y.o. (HR = 0.50, 95% CI: 0.26–0.96, *p* = 0.036), T stage ≤ 2c (HR = 0.34, 95% CI: 0.15–0.77, *p* = 0.009), Number of NCCN high risk criteria = 1 (HR = 0.27, 95% CI: 0.12–0.61, *p* = 0.002), and pre-RT PSA ≤ 0.2 ng/ml (HR = 0.48, 95% CI: 0.26–0.91, *p* = 0.024) were associated with superior overall survival. Initial PSA ≤ 20 ng/ml, Gleason score ≤ 7, NAADT duration ≤ 6 months, and combination with anti-androgen were not relevant to overall survival. Significant variables in the univariate analysis were further studied in a multivariate cox regression analysis. Age ≤ 70 y.o. (HR = 0.51, 95% CI: 0.27–0.99, *p* = 0.046) and pre-RT PSA < 0.2 ng/ml (HR = 0.49, 95% CI: 0.26–0.94, *p* = 0.032) were independent variables associated with better OS (Table 3).

**Figure 1:**
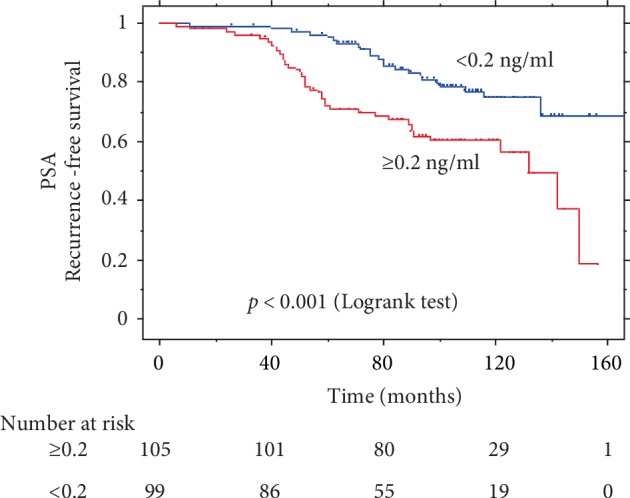
Kaplan–Meier survival curve of PSA-PFS by pre-RT PSA.

**Figure 2:**
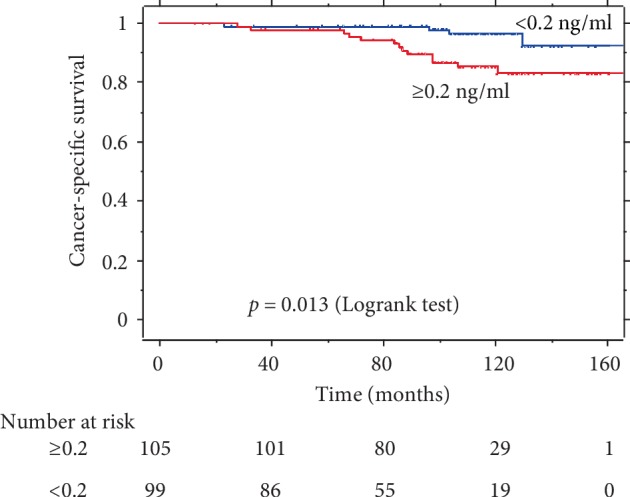
Kaplan–Meier survival curve of CSS by pre-RT PSA.

**Figure 3:**
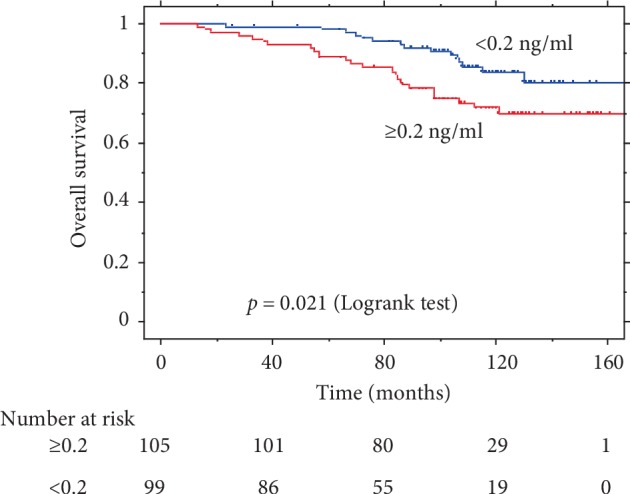
Kaplan–Meier survival curve of OS by pre-RT PSA.

Comparisons of PSA-PFS, CSS, and OS rates according to pre-RT PSA levels are presented in Figures 1, 2, and 3, respectively. Pre-RT PSA less than 0.2 ng/ml was significantly associated with better outcomes in PSA-PFS (HR = 0.42, 95% CI: 0.25–0.70, *p* = 0.001), CSS (HR = 0.27, 95% CI: 0.09–0.82, *p* = 0.013), and OS (HR = 0.48, 95% CI: 0.26–0.91, *p* = 0.021).

## 4. Discussion

Several clinical trials in the past years indicated the effectiveness of EBRT plus ADT for high risk localized prostate cancer. The results of RTOG86-10 and TROG9610 indicated the improvement of PSA progression-free survival, cancer-specific survival and overall survival in a patient treated with neoadjuvant ADT. Other randomized control studies (EORTC22863, RTOG85-31, RTOG92-02) regarding adjuvant ADT more than 2 years, showed significant longer overall survival in patient with locally advanced prostate cancer. In those studies, 5-years PSA-PFS and OS were 82.0–85.2%, 84.3–92.3%, 10-years PSA-PFS and OS were 58.4–71.4% and 67.0–80.2%, respectively. In our study, 5-years PSA-PFS, OS were 84.1%, 93.6%, and 10 years PSA-PFS and OS were 67.9% and 78.1%, respectively, which were comparable to the other reports.

In our institution, irradiation dose was determined by the radiologist 70 Gy/35 fr, and they do not perform dose escalation. Regarding dose of irradiation, the benefit of high dose irradiation of 74 Gy or more has been reported in recent years (PROG95-09 [15], MRC RT01 [16], MDACC [17]). Zelefsky et al. reported long recurrence free survival of high dose EBRT (dose escalation from 70.2 Gy to 89.4 Gy) in 2,551 patients with prostate cancer [18]. Furthermore, Nguyen et al. reported that significant better PSA-PFS and OS in the patients treated with high-doses EBRT of 75.6 Gy or more [19]. However, the high-dose irradiation seems to raise the frequency of severe adverse event related to genitourinary and gastrointestinal disorders [20, 21]. In addition, recent large RCT revealed dose escalation did not improve OS, despite improvements in PSA failure and distant metastasis [22]. In the present study, all 204 patients were treated with 70 Gy irradiation, and no patients had adverse events of grade 3 or more in the long follow-up period. From these results, it is considered that 70 Gy irradiation is tolerable for the patient with high-risk prostate cancer treated with ADT.

We assessed the relationship between clinicopathological factors and PSA recurrence, disease-specific death, and all-cause death. In univariate analysis, age, T stage, number of NCCN high-risk criteria, pre-RT PSA were prognostic factors. In multivariate analysis, age < 70 y.o. and pre-RT < 0.2 ng/ml were associated with significant good prognosis. Several reports showed that PSA before radiation therapy predicts good PSA recurrence-free survival, cancer-specific survival and overall survival. Zelefsky et al. conducted a retrospective analysis of the pre-RT PSA values in 1045 patients treated with ADT and EBRT. The 10-year PSA-RFS was 74.3% in good responders (pre-RT PSA < 0.3 ng/ml) compared to 57.7% in patients with higher PSA nadir values (*p* < 0.001) [9]. Alexander et al. also reported that pre-RT PSA < 0.1 ng/ml was associated better PSA-RFS, CSS, and OS in patients with localized prostate cancer and iPSA more than 40 ng/ml (*n* = 64) [10]. Additionally, Ludgate et al. analyzed 407 patients with NCCN high-risk prostate cancer and reported that patients with PSA nadir before irradiation less than 0.1 ng/ml had long cancer-specific survival [11]. Pre-RT PSA value during NADT may be an indicator to determine local disease control and suppression of micro-metastasis. Therefore, lower pre-RT PSA can predict favorable prognosis in patients with high risk localized prostate cancer.

However, there is no evidence that oncologic outcome can be improved by the prolongation of NADT or combined androgen blockade with anti-androgens before irradiation. In the present study, the duration of NADT was not associated with better prognosis. The above-mentioned study by Ludgate et al. reported that the duration of the NADT and total ADT were not associated with better PSA-RFS, CSS, or OS [11]. In our data, also, the duration of NADT was not associated with better prognosis in multivariate analysis. Furthermore, patients achieved lower pre-RT PSA were more likely to be treated with anti-androgens, however, combined androgen blockade was not associated with superior outcome. Thus, intensive treatment to reduce PSA value may not have high efficacy in the cancer control before irradiation. Overall survival of patients showed slow PSA response cannot be improved by unnecessarily long-term NADT or the addition of anti-androgen.

Therefore, PSA response to primary NADT is an indicator for determining whether a more powerful treatment should be selected. In other words, patients who could not achieve abovementioned minimum PSA levels after NADT should be followed up carefully, and advanced treatment may be considered for them.

Recently, many reports have shown effectiveness of tri-modality therapy using brachytherapy, EBRT, and ADT in combination for patients with high-risk prostate cancer [23, 24]. Furthermore, ASCEND-RT trial data showed that, low-dose-rate brachytherapy boost with whole pelvic irradiation has superior potential compared with dose escalated EBRT in patients with intermediate- and high-risk prostate patients [25]. Induction of the curative modalities possibly improve the prognosis of patients whose pre-RT PSA high before irradiation. Predicting endogenous radiosensitivity early after the initiation of ADT may enable individualized treatment in selected prostate cancer patients at high risk. Accordingly, significant decline of PSA with neoadjuvant ADT is an important indicator for selecting more intensive therapy for patient with high risk localized prostate cancer.

In the interpretation of the current study's results, there are several limitations. First, this study is a single center retrospective cohort, therefore, it includes a relatively small sample size and the biases in patient and treatment selection. Second, total RT doses used in modern practice are generally higher than those used during the era of this study from 2005 to 2010. Therefore, it is controversial whether it can adapt to the current standard RT and dose escalated RT.

## 5. Conclusion

In conclusion, achieving pre-RT PSA < 0.2 ng/ml, rather than patient characteristics, was significantly associated with superior PSA recurrence-free survival, cancer-specific survival, and overall survival in patients with high-risk locally advanced prostate cancer treated with ADT and RT. If the pre-RT PSA does not reach low levels, combination therapy with more powerful modality such as brachytherapy could improve treatment outcome.

## Figures and Tables

**Table 1:** Patient and treatment characteristics, compared between patients with a pre-RT PSA <0.2 ng/ml and ≥0.2 ng/ml.

Variables	All	Pre-RT PSA < 0.2	Pre-RT PSA ≥ 0.2
	(*n* = 204)	(*n* = 105)	(*n* = 99)
Age [y.o.], median (range)	71 (57–83)	71 (57–83)	71 (58–82)
Initial PSA [ng/ml], median (range)	24.7 (1.9–282.5)	20.3 (1.9–268)	31.0 (5.33–282.5)

*Clinical T stage, n (%)*			
T1	36 (17.7)	18 (17.1)	18 (8.2)
T2	38 (18.6)	17 (16.2)	21 (21.2)
T3	123 (60.3)	68 (64.8)	55 (55.6)
T4	7 (3.4)	2 (1.9)	5 (5.0)
*Gleason score, n (%)*			
			
7 or less	73 (35.8)	34 (32.4)	39 (39.3)
8 or higher	131 (64.2)	71 (67.6)	60 (60.7)
			
*No. NCCN high risk factor, n (%)*			
1	85 (41.7)	45 (42.9)	40 (40.4)
2 or 3	119 (58.3)	60 (57.1)	59 (59.6)
			
*LH-RH agonist + bicalutamide, n (%)*			
Yes	142 (69.6)	95 (90.5)	47 (47.5)
No	62 (30.4)	10 (95)	52 (52.5)
Follow-up period [mo], median (IQR)	113 (95–128)		
Neoadjuvant HT [mo], median (IQR)	7 (6–10)		
Total HT [mo], median (IQR)	27 (14–38)		
PSA before RT [ng/ml], median (range)	0.20 (0.01–22.1)		

**Table 2:** Multivariate analysis of factors affecting PSA recurrence-free survival.

Variables	Univariate analysis		Multivariate analysis	
	HR (95% CI)	*p*-value	HR (95% CI)	*p*-value
Age ≤ 70 y.o.	1.167 (0.707–1.926)	0.546		
Initial PSA ≤ 20 ng/ml	0.610 (0.255–1.050)	0.074	0.795 (0.501–1.261)	0.329
T stage ≤ T2c	0.673 (0.292–1.156)	0.151		
Gleason score ≤ 7	0.581 (0.332–0.018)	0.058	0.872 (0.594–1.281)	0.485
NCCN risk factor = 1	0.423 (0.242–0.741)	0.003	1.249 (0.766–2.036)	0.374
Anti-androgen +	0.602 (0.360–1.005)	0.052	1.036 (0.663–1.621)	0.876
Pre-RT PSA < 0.2 ng/ml	0.418 (0.249–0.702)	0.001	0.848 (0.580–1.240)	0.394
NAHT ≤ 6mo	0.730 (0.428–1.244)	0.247		

**Table 3:** Multivariate analysis of factors affecting overall survival.

Variables	Univariate analysis		Multivariate analysis	
	HR (95% CI)	*p*-value	HR (95% CI)	*p*-value
Age ≤ 70 y.o.	0.503 (0.264–0.955)	0.036	0.514 (0.267–0.990)	0.046
Initial PSA ≤ 20 ng/ml	0.565 (0.284–1.124)	0.104		
T stage ≤ T2c	0.340 (0.151–0.765)	0.009	0.441 (0.149–1.307)	0.140
Gleason score ≤ 7	0.506 (0.249–1.031)	0.061	0.568 (0.259–1.248)	0.159
NCCN risk factor = 1	0.268 (0.119–0.605)	0.002	0.543 (0.174–1.698)	0.294
Anti-androgen +	1.164 (0.596–2.274)	0.657		
Pre-RT PSA < 0.2 ng/ml	0.484 (0.257–0.910)	0.024	0.493 (0.258–0.941)	0.032
NAHT ≤ 6mo	0.916 (0.486–1.723)	0.784		

## Data Availability

The data used to support the findings of this study are available from the corresponding author upon request.

## References

[1] Roach M., Bae K., Speight J. (2008). Short-term neoadjuvant androgen deprivation therapy and external-beam radiotherapy for locally advanced prostate cancer: long-term results of RTOG 8610. *Journal of Clinical Oncology*.

[2] Denham J. W., Steigler A., Lamb D. S. (2005). Short-term androgen deprivation and radiotherapy for locally advanced prostate cancer: results from the trans-tasman radiation oncology group 96.01 randomised controlled trial. *The Lancet Oncology*.

[3] Bolla M., Collette L., Blank L. (2002). Long-term results with immediate androgen suppression and external irradiation in patients with locally advanced prostate cancer (an EORTC study): a phase III randomized trial. *Lancet*.

[4] Pilepich M. V., Caplan R., Byhardt R. W. (2002). Phase III trial of androgen suppression using goserelin in unfavorable-prognosis carcinoma of the prostate treated with definitive radiotherapy: report of radiation therapy oncology group protocol 85–31. *Journal of Clinical Oncology*.

[5] Horwitz E. M., Bae K., Hanks G. E. (2008). Ten-year follow-up of radiation therapy oncology group protocol 92–02: a phase III trial of the duration of elective androgen deprivation in locally advanced prostate cancer. *Journal of Clinical Oncology*.

[6] Roach M. (1997). Neoadjuvant therapy prior to radiotherapy for clinically localized prostate cancer. *European Urology*.

[7] Joon D. L., Hasegawa M., Sikes C. (1997). Supraadditive apoptotic response of R3327-G rat prostate tumors to androgen ablation and radiation. *International Journal of Radiation Biology*.

[8] Zilli T., Dal Pra A., Kountouri M., Miralbell R. (2016). Prognostic value of biochemical response to neoadjuvant androgen deprivation before external beam radiotherapy for prostate cancer: a systematic review of the literature. *Cancer Treatment Reviews*.

[9] Zelefsky M. J., Gomez D. R., Polkinghorn W. R., Pei X., Kollmeier M. (2013). Biochemical response to androgen deprivation therapy before external beam radiation therapy predicts long-term prostate cancer survival outcomes. *International Journal of Radiation Oncology, Biology, Physics*.

[10] Alexander A., Crook J., Jones S. (2010). Is biochemical response more important than duration of neoadjuvant hormone therapy before radiotherapy for clinically localized prostate cancer? an analysis of the 3-versus 8-month randomized trial. *International Journal of Radiation Oncology, Biology, Physics*.

[11] Ludgate C. M., Bishop D. C., Pai H. (2005). Neoadjuvant hormone therapy and external-beam radiation for localized high-risk prostate cancer: the importance of PSA nadir before radiation. *International Journal of Radiation Oncology, Biology, Physics*.

[12] McGuire S. E., Lee A. K., Cerne J. Z. (2013). PSA response to neoadjuvant androgen deprivation therapy is a strong independent predictor of survival in high-risk prostate cancer in the dose-escalated radiation therapy era. *International Journal of Radiation Oncology, Biology, Physics*.

[13] Roach M., Hanks G., Thames Jr. H. (2006). Defining biochemical failure following radiotherapy with or without hormonal therapy in men with clinically localized prostate cancer: recommendations of the RTOG-ASTRO Phoenix Consensus Conference. *International Journal of Radiation Oncology, Biology, Physics*.

[14] National Cancer InstituteNational Institutes of Health (2017). Common Terminology Criteria for Adverse Events (CTCAE), version 5.0.

[15] Zietman A. L., Bae K., Slater J. D. (2010). Randomized trial comparing conventional-dose with high-dose conformal radiation therapy in early-stage adenocarcinoma of the prostate: long-term results from proton radiation oncology group/American college of radiology 95-09. *Journal of Clinical Oncology*.

[16] Dearnaley D. P., Sydes M. R., Graham J. D. (2007). Escalated-dose versus standard-dose conformal radiotherapy in prostate cancer: first results from the MRC RT01 randomised controlled trial. *The Lancet Oncology*.

[17] Pollack A., Zagars G. K., Starkschall G. (2002). Prostate cancer radiation dose response: results of the MD Anderson phase III randomized trial. *International Journal of Radiation Oncology, Biology, Physics*.

[18] Zelefsky M. J., Pei X., Chou J. F. (2011). Dose escalation for prostate cancer radiotherapy: predictors of long-term biochemical tumor control and distant metastases–free survival outcomes. *European Urology*.

[19] Nguyen Q. N., Levy L. B., Lee A. K. (2013). Long-term outcomes for men with high-risk prostate cancer treated definitively with external beam radiotherapy with or without androgen deprivation. *Cancer*.

[20] Pollack A., Zagars G. K., Smith L. G. (2000). Preliminary results of a randomized radiotherapy dose-escalation study comparing 70 Gy with 78 Gy for prostate cancer. *Journal of Clinical Oncology*.

[21] Al-Mamgani A., van Putten W. L., Heemsbergen W. D. (2008). Update of Dutch multicenter dose-escalation trial of radiotherapy for localized prostate cancer. *International Journal of Radiation Oncology, Biology, Physics*.

[22] Michalski J. M., Moughan J., Purdy J. (2018). Effect of standard vs dose-escalated radiation therapy for patients with intermediate-risk prostate cancer. *JAMA Oncology*.

[23] Stock R. G., Cesaretti J. A., Hall S. J., Stone N. N. (2009). Outcomes for patients with high-grade prostate cancer treated with a combination of brachytherapy, external beam radiotherapy and hormonal therapy. *BJU International*.

[24] Ennis R. D., Hu L., Ryemon S. N., Lin J., Mazumdar M. (2018). Brachytherapy-based radiotherapy and radical prostatectomy are associated with similar survival in high-risk localized prostate cancer. *Journal of Clinical Oncology*.

[25] Morris W. J., Tyldesley S., Rodda S. (2017). Androgen suppression combined with elective nodal and dose escalated radiation therapy (the ASCENDE-RT Trial): an analysis of survival endpoints for a randomized trial comparing a low-dose-rate brachytherapy boost to a dose-escalated external beam boost for high-and intermediate-risk prostate cancer. *International Journal of Radiation Oncology, Biology, Physics*.

